# Awareness, attitudes and practices for osteoporosis prevention among postmenopausal women: A mixed-method survey and DEXA correlation

**DOI:** 10.6026/973206300221991

**Published:** 2026-04-30

**Authors:** Sanjay M Walulkar

**Affiliations:** 1 Department of Anatomy, NKP Salve Institute Medical Sciences and Research center and Lata Mangeshkar Hospital Nagpur, India

**Keywords:** Osteoporosis, postmenopausal women, awareness, attitudes, preventive practices, bone mineral density, DEXA scan, health education, risk factors, osteoporosis prevention

## Abstract

Osteoporosis remains under-recognized among postmenopausal women despite its strong association with fracture risk and long-term disability. Therefore, it is of interest evaluate awareness, attitudes and preventive practices and correlated them with bone mineral density using DEXA in 200 postmenopausal women. Low bone mass was highly prevalent, with 46% osteopenia and 25% osteoporosis, while only 39% demonstrated adequate awareness and preventive practices were limited. Higher awareness and regular preventive behaviors were significantly associated with normal bone mineral density and older age, lower BMI, poor awareness and lack of exercise predicted low bone density. Thus, strengthening education, screening access and lifestyle interventions is essential to improve bone health outcomes in this population.

## Background:

Osteoporosis is a progressive skeletal disease with decreased bone density (mass) and increased susceptibility to fractures, worldwide, primarily in post-menopausal women due to the absence of estrogen [1]. Osteoporosis often does not become symptomatic until there is a fracture, thus early detection and prevention are paramount in the prevention of the disease [2]. Despite the increasing global impact of osteoporosis, there is insufficient knowledge of the disease and its prevention in numerous worldwide populations [3]. A variety of lifestyle factors contribute greatly to poor bone health and osteoporosis, including very low calcium intake, low vitamin D levels and a sedentary lifestyle [4]. Dual-energy x-ray absorptiometry (DEXA) is currently accepted as the standard method of evaluating bone mineral density and identifying individuals who are at a higher risk for developing osteoporosis [5]. Unfortunately, due to the limited availability of testing or lack of knowledge about the disease, many women do not receive proper diagnostic or preventive services [6]. Recent research has indicated that evaluating how women understand and perceive their bone health may assist in developing targeted community-based intervention programs [7]. Therefore, it is of interest to evaluate postmenopausal women's awareness, attitudes and preventive practices regarding the prevention of osteoporosis and their correlation with DEXA results.

## Materials and Methods:

In between the months of March and August in 2025, this mixed method cross sectional study was conducted on post-menopausal women attending outpatient/community health clinics of a tertiary hospital. To be considered for inclusion in the study, participants were to be between the ages of 45 and 65 and have been naturally menopausal for a minimum of 12 months. Inclusion was determined through a process of consecutive sampling only (out of 200 women who met the age requirement, only those who had not been diagnosed with any of the listed exclusion criteria were eligible to participate). To ensure ethical research was conducted, ethical approval was obtained and written informed consent was provided by each participant prior to participating. Data was collected from the sample using Form A, a structured, pre-tested questionnaire that contained data on sociodemographic, reproductive history, awareness of osteoporosis risk factors, attitudes towards prevention and self-reported behaviour towards prevention through means of diet, exercise and sunlight exposure through use of vitamin D supplementation. A purposive subsample of 20 participants from the purposively sampled participants were interviewed using semi-structured interviews in order to gain understanding of barriers to engaging in preventative behaviours and motivating factors to engage in preventative behaviours. All study participants were assessed via DEXA scanning procedures performed by trained technicians. Scanning measured L1 to L4 spinal region, as well as femoral neck, of each study participant. Each T-score was classified according to World Health Organisation classification (normal, osteopenia or osteoporotic). All anthropometric and clinical data were collected during t he assessment phase of study. Quantitative data were analysed descriptively using frequencies, chi-square, t-tests and ANOVA, which was then analysed using statistical modelling (multivariable regression) to determine predictors of low bone mineral density. Qualitative data were analysed thematically. A p-value of < 0.05 was used to determine level of statistical significance.

## Results:

In this study, we found 200 women who had all been through menopause, with a mean (standard deviation) age of (59.8±6.7) and an average BMI of (27.2 ±4.1) kg/m^2^. We measured their bone density using dual energy x-ray absorptiometry (DEXA) and found that 46% had osteopenia; 25% were diagnosed with osteoporosis, while the majority (71%) of these postmenopausal women had a low bone density compared to the norm. There was limited global awareness about osteoporosis amongst postmenopausal women, with only 39% having adequate knowledge about the disease and only 29% aware that DEXA scans exist as a means for assessing bone density. However, despite the fact that 71% of the sample recognized osteoporosis to be an important health concern, they reported inconsistent preventive behaviors; 33% of the sample exercised regularly; only 46% consumed sufficient quantities of calcium; 42% received adequate amounts sunlight exposure. Postmenopausal women with normal bone density had significantly more awareness of osteoporosis and more regular preventive behaviors than those without normal bone density (p<0.05). There was a strong positive correlation between women's attitudes towards osteoporosis and their actual practices regarding osteoporosis prevention (r=0.72; p<0.001). Multivariable regression analysis revealed that higher age, lower BMI, poor knowledge about osteoporosis and a lack of exercise were independent predictors of having low bone density. Qualitative data supported the quantitative findings; i.e., the low levels of knowledge about osteoporosis; inaccessibility of screenings; and cultural issues were perceived by women to be the primary barriers to preventing osteoporosis. Table 1 (see PDF) provides baseline characteristics of 200 postmenopausal women, showing a mean age of 59.8 years, mean BMI of 27.2 kg/m^2^ and most being homemakers with moderate education. Table 2 (see PDF) demonstrates bone mineral density distribution, with 46% having osteopenia and 25% osteoporosis, indicating widespread low bone mass. Table 3 (see PDF) shows limited awareness, as only 39% had adequate knowledge, especially regarding exercise and DEXA screening. Table 4 (see PDF) presents attitudes, revealing that most women viewed osteoporosis as serious and preventable but fewer emphasized routine screening. Table 5 (see PDF) provides data on preventive practices, showing low engagement in exercise, calcium intake and sun exposure. Table 6 (see PDF) compares awareness and BMD, demonstrating significantly better bone health among women with higher awareness (p < 0.001). Table 7 (see PDF) shows that regular calcium intake, sun exposure and exercise were strongly linked to normal BMD. Table 8 (see PDF) demonstrates a strong positive correlation (r = 0.72, p < 0.001) between positive attitudes and preventive practices. Table 9 (see PDF) identifies predictors of low BMD older age, lower BMI, poor awareness and lack of exercise increased risk significantly. Finally, Table 10 (see PDF) highlights qualitative insights, identifying poor awareness, cultural barriers, limited screening access and the positive influence of education on preventive behavior.

## Discussion:

The findings from the current study indicate that low bone mass levels are prevalent in postmenopausal women and that there is major knowledge gaps regarding prevention of this condition [8]. Approximately 75% of those studied had either osteopenia or osteoporosis, which corresponds to recent studies demonstrating an increased prevalence of osteoporosis in older adults [9]. Although three out of four women shown in the current research had some form of osteoporosis, less than 40% had adequate knowledge, which is consistent with reports on the continuing gap in global vigilance regarding osteoporosis [10]. While many of the participants thought that osteoporosis was a serious health problem, there was little engagement by the participants with regards to prevention practices (e.g., exercise, dietary calcium, sunlight) [11]. The discrepancy between knowledge and the execution of preventive behaviour has been reported in previous community research [12]. Awareness of osteoporosis, preventive behaviour and BMD are all related and emphasise the need for health education programs that promote improved bone health [13]. Participating in a healthy lifestyle, including lower age, BMI and exercise has been shown to be associated with decreased incidence of low BMD as established risk factors [14]. The qualitative survey revealed limited access to screening, cultural influences with respect to nutrition and inadequate physician recommendations for preventative intervention for osteoporosis as identified barriers to preventive practice [15]. This highlights the importance of culturally-targeted education and accessible means of screening to address the barriers to preventive behaviours [16]. Developing and integrating osteoporosis education and DEXA screening into standard medical practice during routine visits to primary and/or gynaecological care providers may facilitate early identification and the adoption of preventive health behaviours [17]. In addition, community-based interventions promoting healthy nutrition and physical activity combined with routine screening for osteoporosis will reduce the risk of fractures and improve long-term quality of life among postmenopausal women.

## Conclusion:

The low amount of bone density in postmenopausal females occurs frequently and has been discovered to be characterized by an extremely low level of knowledge or understanding associated with the prevention of low bone mass. Formal educational efforts, promotion of lifestyle changes and access to screening will help minimize the risk of developing osteoporosis through improved long-term health of bones.
